# The Effect of TNF and VEGF on the Properties of Ea.hy926 Endothelial Cells in a Model of Multi-Cellular Spheroids

**Published:** 2018

**Authors:** S. Sh. Gapizov, L. E. Petrovskaya, L. N. Shingarova, E. V. Svirschevskaya, D. A. Dolgikh, M. P. Kirpichnikov

**Affiliations:** Shemyakin–Ovchinnikov Institute of Bioorganic Chemistry, Russian Academy of Sciences, Miklukho-Maklaya Str. 16/10, Moscow, 117997, Russia; Lomonosov Moscow State University, Department of Biology, Leninskie Gory 1, bldg. 12, Moscow, 119234, Russia

**Keywords:** 2D and 3D cultures, αvβ3-integrin, vascular endothelial growth factor receptor 2, intercellular adhesion molecule, tumor necrosis factor, inflammation

## Abstract

Endothelial cells play a major role in the development of inflammation and neoangiogenesis in cancer and chronic inflammatory diseases. In 3D cultures, cells are under conditions that closely resemble those existing in healthy and disease-stricken human organs and tissues. Therefore, the development of a 3D model based on the Ea.hy926 endothelial cell line is an urgent need in molecular and cellular biology. Cell cultivation on an anti-adhesive substrate under static conditions was shown to lead to the formation of spheroids (3D cultures). Expression of ICAM-1 and VEGFR-2 and production of cytokines were screened in 2D and 3D cultures in the presence of TNF and VEGF. According to flow cytometry and confocal microscopy data, TNF significantly increased the expression of the cell adhesion molecule ICAM-1 in both 2D and 3D cultures but did not affect the expression level of VEGFR-2. Increased production of pro-inflammatory (IL-8, IL-6, IP-10) and anti-inflammatory (IL-10, TGF-β 1–3) factors was observed in spontaneous 3D cultures but not in 2D cultures, which was confirmed by flow cytometry and qPCR. TNF-induced secretion of IL-10, GM-CSF, and IL-6 was 11-, 4.7-, and 1.6-fold higher, respectively, in 3D cultures compared to 2D cultures. Thus, the use of a Ea.hy926 3D cell culture is a promising approach in studying the effects of anti- and pro-inflammatory agents on endothelial cells.

## INTRODUCTION

Cancer and chronic inflammatory diseases involving various human organs and tissues are a serious medical and societal problem. The tumor necrosis factor alpha (TNF-α) has been shown to play the key role in the development and maintenance of inflammation in diseases, such as rheumatoid arthritis, psoriasis, Crohn’s disease, etc. [1, 2]. Both the inflammatory process and tumor growth are accompanied by tissue hypoxia, which leads to the formation of new blood vessels under the influence of the vascular endothelial growth factor (VEGF), which is secreted by epithelial cells in conditions of hypoxia [3, 4]. The expression level of αvβ3-integrin in endothelial cells is known to be significantly increased in tumor vessels [5]. In endothelial cells, TNF and VEGF have been shown to stimulate the expression of adhesion and inflammation molecules, in particular ICAM-1 and VCAM-1, the vascular endothelial growth factor receptor 2 (VEGFR-2), PECAM-1, and P- and E-selectins, induce the release of the von Willebrand factor from Weibel-Palade bodies, as well as enhance the secretion of the cytokines IL-6 and IL-8, monocyte chemotaxis protein 1 (MCP-1), and the granulocyte- macrophage colony-stimulating factor (GM-CSF) [6-11]. A change in the expression of endothelial surface proteins ensures the inhibition of leukocytes at the site of an inflammation, as well as their adhesion and transendothelial migration [12]. The *in vitro* response corresponds to the *in vivo *processes occurring under the influence of pro-inflammatory stimuli, which makes it possible to use an endothelial cell culture to simulate inflammation processes in the body.

The use of therapeutic agents capable of suppressing angiogenesis partially slows down the pathological process. In particular, anti-VEGF antibodies (Bevacizumab), a low-molecular-weight inhibitor of VEGF (Aflibercept), anti-TNF antibodies (Adalimumab, Infliximab, and Etanercept), and a number of anti-integrin antibodies (Vedolizumab and anti-α4β7 integrin antibodies) have been developed and clinically used [13-15]. The αvβ3-integrin inhibitor known as Cilengitide, the antibodies Etaracizumab, and other drugs are undergoing clinical trials [16-18]. The disadvantage of low-molecular-weight drugs is that the patient quickly develops resistance to them [19]. Antibodies also have a number of disadvantages; in particular, the high cost of production of humanized recombinant antibodies limits the number of patients who can afford the therapy. On the other hand, antibodies have a large molecular weight, which prevents their deep penetration into tissues [19, 20].

The development of antibody analogues for creating immunoconjugates with antitumor drugs and/or vascular growth inhibitors will improve the treatment of tumor and chronic inflammatory diseases and expand the range of patients that can receive adequate therapy [19]. Primary screening of new drugs requires an *in vitro* cell model with properties that are as close as possible to *in vivo* conditions. Currently, interactions between anti-inflammatory drugs and endothelial cells are analyzed using primary cultures derived from the umbilical vein of healthy donors (human umbilical vein endothelial cells, HUVECs) or the Ea.hy926 hybrid line [21-23]. It is preferable to use a stable line, because the functional characteristics of HUVECs may depend on the quality of cell isolation and on the donor; in addition, donor cells are not always available and the number of passages of primary cells is limited [24]. The functional characteristics of HUVECs and Ea.hy926 largely coincide; in particular, both cell types change the expression of adhesion molecules and production of IL-6 and IL-8 in response to TNF [25-27].

In the body, small vessels and capillaries are predominantly composed of endotheliocytes; in larger vessels, the wall is formed by endothelial cells, connective tissue, and smooth muscles. A monoculture of endothelial cells largely simulates the capillary structure; in this case, the use of multicellular spheroids of endothelial cells enables one to study the effects of various drugs not only on endothelial cells, but also on their associates with the connective matrix formed in 3D cultures [28-31]. Earlier, there have been attempts to develop 3D cultures of endothelial cells by clinostatting [32-35]. This method is based on rotating a cell culture in a gravity field, which leads to the formation of spheroids on the monolayer culture surface. The purpose of the present work was to develop a static 3D culture model based on the Ea.hy926 endothelial cell line and compare the responses of 2D and 3D cultures to TNF and VEGF.

## EXPERIMENTAL

Reagents from Bio-Rad (USA), Sigma (USA), Merck (USA), Panreac (Spain), and PanEco (Russia) were used in the study. Solutions were prepared in Milli-Q deionized water. The recombinant proteins TNF (produced in the Laboratory of Protein Engineering of the Institute of Bioorganic Chemistry) and VEGFA165 (Protein Synthesis, Russia) were used.

**Cell cultures**

A human Ea.hy926 endothelial cell line (ATCC, CRL- 2922) provided by A.A. Sokolovskaya (Research Institute of General Pathology and Pathophysiology) with the permission of Dr. C.-J. Edgell (University of North Carolina) was used in the study. Cells were incubated in a DMEM/F12 medium (PanEco, Russia) supplemented with 10% inactivated bovine fetal serum (HyClon, USA), 50 μg/mL gentamicin sulfate, and 2 mM *L*-glutamine (PanEco). To form three-dimensional cultures, the well surface of a 24-well plate (Costar) was coated with poly-2-hydroxyethyl methacrylate (pHEMA) (Sigma). Each well was seeded with 5 × 10^5^ cells per 1 mL of the growth medium. The cells were cultured under standard conditions in a CO_2_ incubator for 48 h until the formation of a confluent monolayer (2D culture) or spheroids (3D culture).

**Confocal microscopy**

To analyze the expression of surface adhesion molecules in the 2D endothelial cell cultures, sterile glass coverslips were placed in six-well plates; 1 × 10^5^ cells in 200 μL of medium were put on each coverslip and incubated in a CO_2_ incubator under standard conditions for 16 h to produce a confluent monolayer. To analyze the 3D cultures of Ea.hy926 cells, spheroids were pipetted and transferred into the wells of a 96-well plate. Cell cultures were added with recombinant TNF or VEGFA proteins to a concentration of 25 ng/mL each and incubated for 5 h. The cells were stained with mouse monoclonal antibodies to human ICAM-1 (CD56) and VEGFR-2 (Flk-1), as well as anti-mouse IgG secondary antibodies labeled with CFL488 (Santa Cruz Biotechnology, USA) or Alexa Fluor 555 (Invitrogen, USA). Antibodies were added to a concentration of 0.2 μg/mL for 1 h. The cells were incubated in a CO_2_ incubator at a rotation speed of 40 rpm. Cell nuclei were stained with Hoechst 33342 (Sigma). After completion of incubation, the 2D and 3D cultures were fixed with 1% paraformaldehyde at room temperature for 10 min and then washed with phosphate buffered saline (PBS). After fixation, the cells were washed from primary antibodies and incubated with secondary antibodies in PBS (1 : 1000 dilution) at 37°C for 40 min. After washing, the cells were polymerized on glass slides using a Mowiol 4.88 (Calbiochem, Germany) medium and left overnight at room temperature. Images were acquired and analyzed using a Nikon Eclipse TE2000-E confocal microscope (Japan).

**Flow cytometry**

The expression of the surface molecules ICAM-1 and VEGFR-2 in all samples was assessed using a FACScan flow cytometer (BD, USA). To prepare a suspension, cells from 2D and 3D cultures were treated with a trypsin/EDTA solution (PanEco), washed in PBS containing 1% bovine serum albumin and 0.05% NaN_3_ (PBSA), combined with the appropriate antibodies, and incubated in the dark at 4°C for 60 min. After washing, the cells were stained with secondary fluorescently labeled antibodies (4°C, in the dark, 60 min). Before the analysis, propidium iodide (0.5 μg/mL) was added to the samples for differential staining of dead cells. In each sample, 10,000 cells were analyzed. The data were analyzed using the WinMDI 2.9 software.

**Production of humoral factors**

Production of cytokines and chemokines by Ea.hy926 cells cultured under 2D and 3D conditions was analyzed by flow cytometry with microparticles on a FACS Calibur instrument (BD, USA) according to the manufacturer’s protocol (BioRad, USA).

**Quantitative PCR (qPCR)**

Total mRNA was isolated using an RNeasy Mini Kit (Qiagen, USA) and purified from DNA contamination by treating it with DNase I (Fermentas, USA). cDNA was synthetized using a First Strand cDNA Synthesis kit (Thermo Scientific, USA). The concentration of mRNA and cDNA was determined using a NanoDrop 2000 device (Thermo Scientific). The resulting cDNA was used as a template for quantitative PCR (qPCR) with specific primers (*Table 1*) [36] and a qPCRmix-HS SYBR mixture (Eurogen, Russia) on a Lightcycler 480 instrument (Roche, USA). The reaction mixture consisted of 50 ng of cDNA, primers (0.120 μM per sample), the qPCRmix-HS SYBR (5x) mixture, and Milli-Q water. The annealing temperature was adjusted in accordance with the primer melting point. The data were sequentially processed using the Convert Light-Cycler 480 and LineRegPCR software. The expression of each gene was analyzed in triplicate.

**Statistics**

The obtained data were analyzed with parametric methods using the Excel software; The Cell Quest software was used for the analysis of flow cytometry data. Differences were considered to be statistically significant at *p* < 0.05.

## RESULTS AND DISCUSSION

**Expression of adhesion molecules by Ea.hy926 cells in 2D and 3D cultures**

**Table 1:** Primers for qPCR [36]

Gene	Primer	Nucleotide sequence, 5’ → 3’	Amplicon size, b.p.	T_m_, °C
β-actin	BA f	TCATGTTTGAGACCTTCAACAC	512	55
	BA r	GTCTTTGCGGATGTCCACG		
GM-CSF	GM f	CTGCTGCTGAGATGAATGAAACAG	195	55
	GM r	GCACAGGAAGTTTCCGGGGT		
ICAM-1	ICAM f	ACCATGGAGCCAATTTCTC	590	51
	ICAM r	ACAATCCCTCTCGTCCAG		
IL-6	IL6 d	GATGCAATAACCACCCCTGACCC	173	52
	IL6 r	CAATCTGAGGTGCCCATGCTAC		
VEGFR-2	VEGFR2 f	ATGCTCAGCAGGATGGCAA	320	53
	VEGFR2 r	TTTGGTTCTGTCTTCCAAAGT		

Normally, the endothelial cells lining the vessels are interconnected by ICAM-1, VCAM-1, and PECAM-1 adhesion molecules, as well as by a number of other actin-associated molecules, which enables the rapid cytoskeletal rearrangement necessary for leukocyte extravasation into tissues during an inflammation [6]. Unlike endothelial cells, epithelial cells are interconnected via tighter cadherin contacts that are linked to the keratin filaments of the cytoskeleton. Epithelial cells form 3D cultures of varying densities, depending on the number of cadherin contacts [37]. Previously, there had been no attempts to produce 3D cultures of endothelial cells which would be similar to epithelial cell cultures. Clinostatted cultures, called 3D-cultures in some works, are monolayer cultures grown by rotation in a gravity field [32-35]. Cultivation for 5 to 6 days results in the development of spheroids on the monolayer surface, which are used for an analysis [33]. However, this prolonged cultivation disables the evaluation of the effects of fast-acting factors: e.g., TNF.

**Fig. 1:**
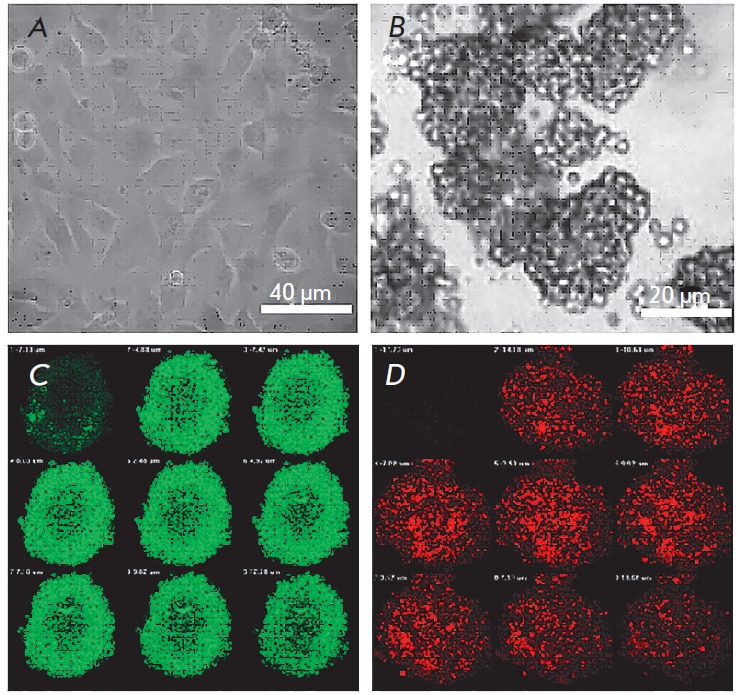
Morphology of Ea.hy926 cells under 2D and 3D cultivation conditions. Ea.hy926 cells 48 h after transfer onto a culture plate (*A*) or a plate with an anti-adhesive coating pHEMA (*B*); light microscopy. Z-stacks of 3D cultures stained with antibodies to ICAM-1 (*C*, green) and VEGFR-2 (*D*, red); confocal microscopy

In this study, Ea.hy926 cells were cultured on an anti- adhesive pHEMA substrate, which resulted in the formation (within 18 h) of 200–400 μm cell clusters indestructible by pipetting (*Fig. 1B*), which confirmed the formation of intercellular contacts throughout the cell surface. In the 2D culture, cells formed a tight monolayer where they formed contacts only along the perimeter (*Fig. 1A*). A confocal analysis of 3D cultures revealed a different expression level of adhesion molecules, depending on the location of the cells in the culture. For example, in Ea.hy926 3D cultures, the level of ICAM-1 expression is higher in cells of the surface layer (*Fig. 1C*), while VEGFR-2 is uniformly expressed by all cells of the spheroid (*Fig. 1D*). Reduced expression of adhesion molecules inside the spheroid is associated with the formation of a hierarchy of cells. The presence of adhesion contacts throughout the cell surface reduces the expression of adhesion molecules – the cell is in the equilibrium state. On the spheroid surface, cells are in contact with the lower layer and have no contacts on the apical surface, which stimulates the expression of adhesion molecules and mimics damage repair in epithelial tissues. Unlike ICAM-1, VEGFR-2 is uniformly expressed throughout the bulk of the spheroid: therefore, endothelial cells, like epithelial cells, proved able of forming internally hierarchical spheroids in static cultures.

**Fig. 2:**
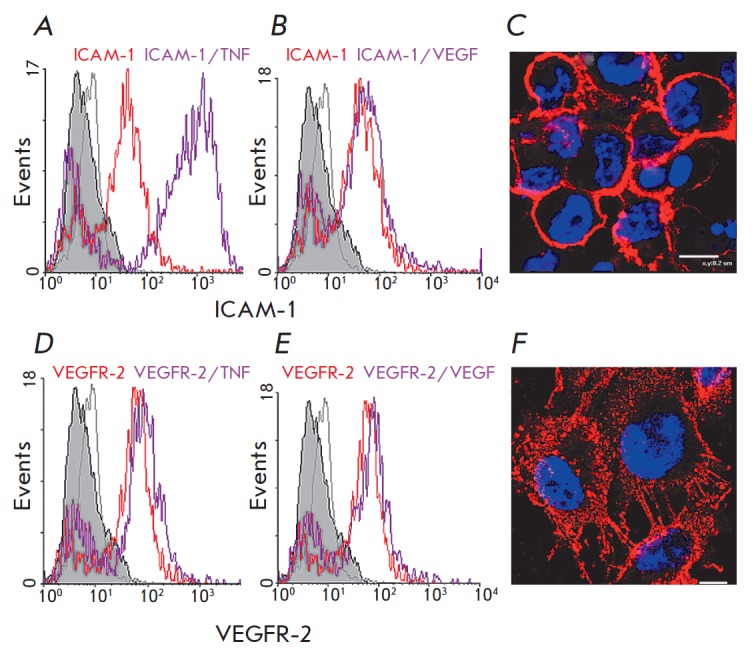
Expression of ICAM-1 and VEGFR-2 in Ea.hy926 cells under the influence of TNF and VEGF A analyzed by confocal microscopy and flow cytometry. *A*, *B*, *D*, *E *– the Y axis is the mean fluorescence intensity; the X axis is the number of events. Ea.hy926 cells were grown under 2D conditions until a confluent monolayer. TNF (*A *and *D*) or VEGF (*B *and *E*) were added (25 ng/mL). The incubation time is 5 hours. Expression of certain proteins by the cells is displayed as fluorescence peaks of antibodies bound to the proteins. The solid gray peak is unstained cells; the gray line is cells with secondary antibodies (negative control); the red line is inactivated cells stained with specific antibodies; the purple line is cells stained with specific antibodies after stimulation by factors. *C *and *F *are representative confocal images of cells stained with antibodies to ICAM-1 (*C*, red) and VEGFR-2 (*F*, red). Cell nuclei are stained with Hoechst 33342 (blue). The scale bar equals C – 8 μm, F – 5 μm

Earlier, studies of Ea.hy926 clinostatted cultures had revealed differences in the expression of adhesion molecules, as well as in spontaneous and TNF-induced cytokine production; in this case, both inhibition [38] and stimulation of the production of several proteins were detected [39]. Expression of adhesion molecules in static 2D and 3D Ea.hy926 cultures in response to TNF and VEGF activation was analyzed using pre-adjusted cell activation conditions. Expression of ICAM-1, VEGFR-2, αvβ3-integrin, and VCAM-1 in the 2D culture under the influence of TNF and VEGF was analyzed by flow cytometry in both early (24 hour incubation) and “old” (72–96 hour incubation) cultures. In addition, the dynamics of the changes in the expression of surface molecules under the influence of factors was studied. There were no changes in the expression of αvβ3-integrin and VCAM-1 (data not shown). VEGF also had no stimulating effect on any of the adhesion molecules. For this reason, the effect of TNF was studied further. TNF was found to act most effectively on early cultures (18–24 h). The effect develops rapidly, achieves a maximum 2–10 h after the addition of TNF, and decreases to control values in 24–36 h. Five hours after the addition of TNF, the expression of ICAM-1 in early cultures increased 13- fold, while the expression of VEGFR-2 remained almost unchanged (*Fig. 2*, *Table 2*). *Figure 2* presents confocal microphotographs of 2D cultures stained with antibodies to ICAM-1 and VEGFR-2 (*Fig. 2 C, F*), which show the typical membrane location of these molecules.

**Fig. 3:**
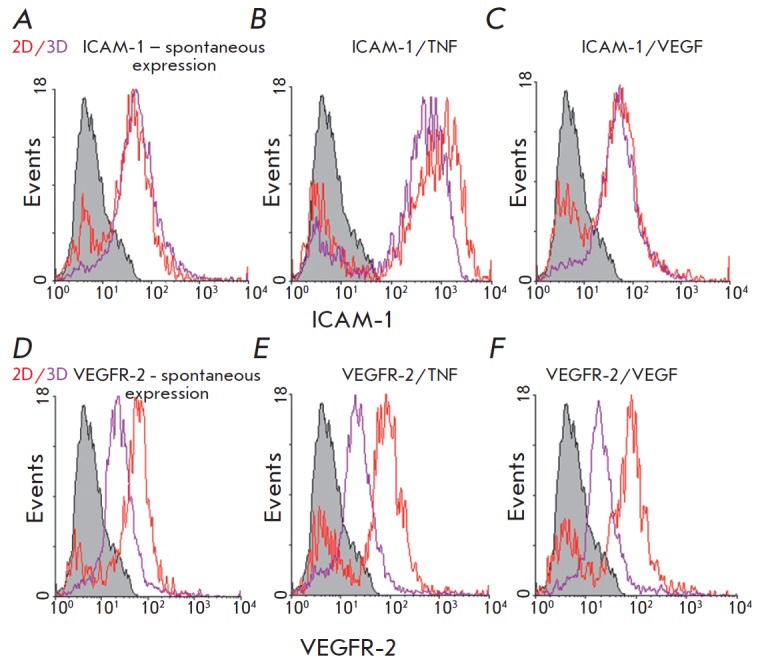
ICAM-1 and VEGFR-2 expression in TNF- or VEGFA-treated Ea.hy926 cells under 2D and 3D conditions compared by flow cytometry. The Y axis is the mean fluorescence intensity; the X axis is the number of events. Ea.hy926 cells were grown under 2D or 3D conditions for 3 days and stained with antibodies to ICAM-1 (*A*–*C*) or VEGFR-2 (*D*–*F*). Cultures were added with TNF (*B*, *E*) or VEGF (*C*, *F*) to a concentration of 25 ng/mL within the last 5 hours. The solid gray peak is the autofluorescence of unstained cells; the red line is the autofluorescence peak of 2D culture cells stained with specific antibodies; the violet line is the autofluorescence peak of 3D culture cells stained in the same way

The comparative data on TNF- and VEGF-induced expression of ICAM-1 and VEGFR-2 in 2D and 3D Ea.hy926 cultures are shown in *Fig. 3*. A more homogeneous pool of cells was shown to form in 3D-cultures. For example, 10–20% of cells in 2D cultures do not express adhesion molecules (a peak in the autofluorescence area), but this parameter is significantly lower (0–5%) in 3D cultures. Unlike ICAM-1, spontaneous expression of VEGFR-2 in 3D cultures is reduced by 50%, despite the absence of the first peak (*Table 2*, *Fig. 3 D*). In all 3D cultures, expression of VEGFR-2 was statistically significantly lower than in 2D cultures, which demonstrates the role of contact interactions in the expression of VEGFR-2 by Ea.hy926 cells.

**Table 2:** Effect of TNF and VEGF on the expression of ICAM-1 and VEGFR-2 by Ea.hy926 cells in 2D and 3D cultures

Culture	Expression	Control	TNF	p	VEGF	p
2D	ICAM-1	49 ± 11	862 ± 148^*^	< 0.001	57 ± 14	> 0.05
3D	ICAM-1	70 ± 15	630 ± 93	< 0.001	63 ± 14	> 0.05
2D	VEGFR-2	59± 11	71 ± 18	> 0.05	67 ± 16	> 0.05
3D	VEGFR-2	32 ± 8^**^	35 ± 8^**^	> 0.05	28 ± 7^**^	> 0.05

The data are presented as relative fluorescence units.

^*^TNF and VEGF were added to a concentration of 25 ng/mL in the last 5 hours of incubation. Expression was assessed by flow cytometry. The effect of a statistically significant increase in TNFα-induced expression compared to the control is shown in bold.

^**^Statistically significant reduction in VEGFR-2 expression in 3D compared to that in 2D.

Expression of ICAM-1 in both 3D and 2D cultures was enhanced by TNF, but the increase was less pronounced in 2D cultures (7- and 11-fold, respectively). In this case, a negative population emerged, like in all 2D cultures (*Fig. 3 B*). VEGF did not affect the expression of adhesion molecules in 3D cultures.

In general, the influence of various factors on the expression level of adhesion molecules in 3D cultures was insignificant compared to that in 2D cultures.

**Production of cytokines by Ea.hy926 cells in 2D and 3D cultures**

**Fig. 4:**
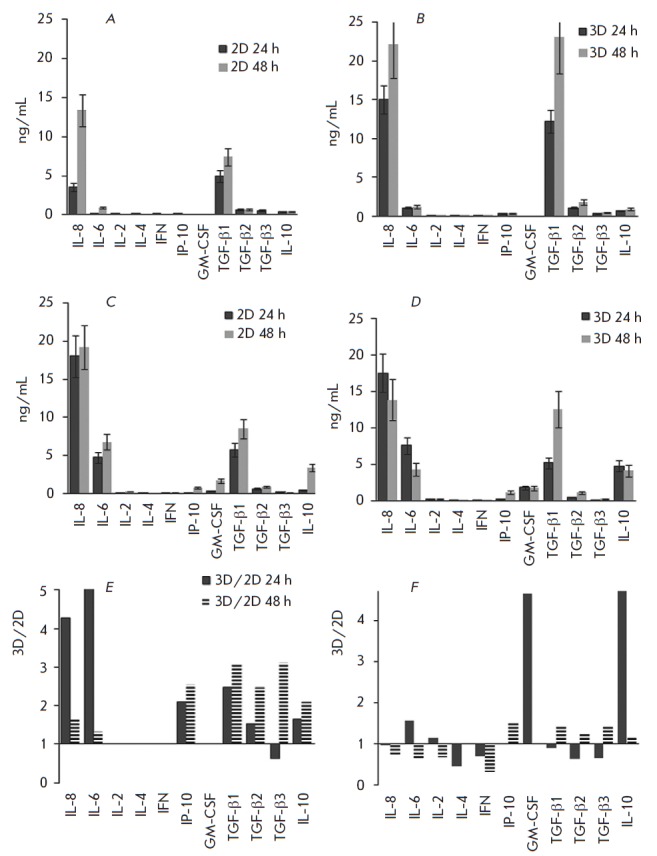
Secretion of humoral factors by Ea.hy926 cells cultured under 2D and 3D conditions. Ea.hy926 cells were cultured in 24-well plates until adhesion or on an anti-adhesive substrate to form 3D cultures. Then, TNF was added to the medium to a concentration of 25 ng/mL. Supernatants were harvested 24 and 48 h after TNF addition. Production of soluble factors in 2D (*A *and *C*) or in 3D (*B *and *D*) cultures without TNF (*A *and *B*) and after TNF addition (*C *and *D*). The ratio of factor concentrations in 3D/2D cultures without stimulation (*E*) and after TNF addition (*F*). The concentration was determined by flow cytometry with microparticles according to the manufacturer’s protocol (BioRad) using calibration curves

One of the indicators of endothelial cell activation is the production of humoral factors: cytokines, chemokines, and growth factors. Because there were no changes in the expression level of adhesion molecules under the influence of VEGF, the production of cytokines in 2D and 3D cultures was analyzed only in the presence of TNF. We analyzed the production of eleven factors, including IL-2, -4, -6, -8, -10, GM-CSF, IFN-γ, transforming growth factors beta (TGF-β) 1–3, and chemokine IP- 10. In the absence of TNF, Ea.hy926 cells were found to produce a significant amount of only IL-8 (13.7g/mL) and TGF-β1 (7.5 ng/mL), with the production in 3D cultures being significantly higher (2- to 3-fold) (*Fig. 4 A, B*). Under the influence of TNF, production of IL-8 in 2D cultures (19 ng/mL) increased to a spontaneous level in 3D cultures (22 ng/mL) and did not change in 3D cultures (*Fig. 4 C, D*). Treatment with TNF resulted in a cytokine production comparable in 2D and 3D cultures, which decreased in the IL-6 > IL-10 > IL-2 > IFN-γ > IL-4 series (*Fig. 4 C, D*). The ratio of spontaneous and TNF-induced production 3D/2D is shown in*Fig. 4 E, F*. Spontaneous 3D cultures produced statistically significantly larger (2- to 5-fold) amounts of IL-8, IL-6, IL-10, TGF-β 1–3, and IP-10, while they almost lacked (below the detection limit in 2D cultures) IL-2, IL-4, IFN-γ, and GM-CSF (*Fig. 4 E*). In TNF-stimulated cultures, the main difference occurred in the production of GM-CSF and IL-10 (*Fig. 4 F*). Secretion of IL-10, GM-CSF, and IL-6 in 3D cultures compared to that in 2D cultures increased 11-, 4.7-, and 1.6-fold, respectively. At the same time, secretion of IL-4, IFN-γ, TGF-β2, and TGF-β3 in 3D cultures compared to that in 2D cultures decreased 2-, 1.4-, 1.6-, and 1.6-fold, respectively (*Fig. 4 F*).

**Comparison of mRNA and protein synthesis by Ea.hy926 cells in 2D and 3D cultures**

**Fig. 5:**
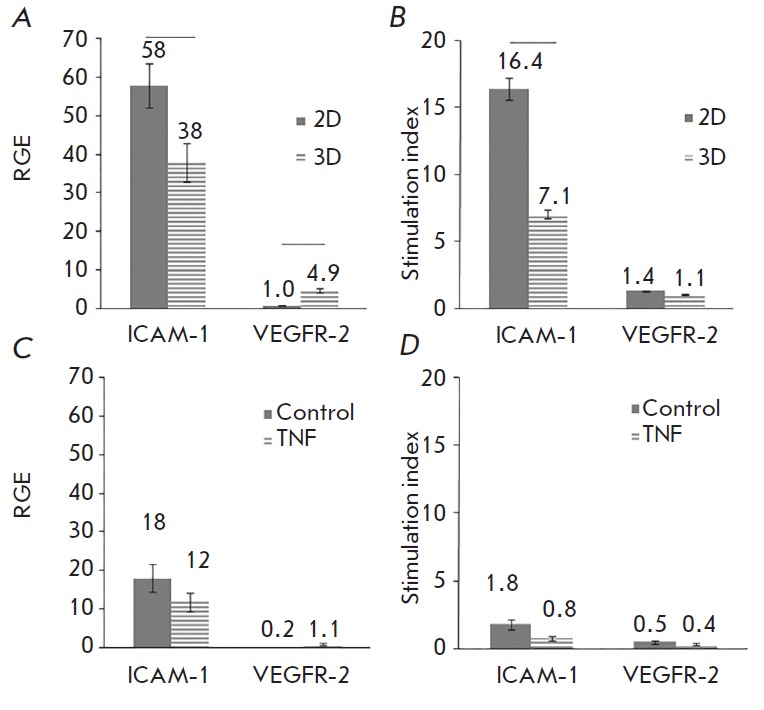
Expression of VEGFR-2 and ICAM-1 in Ea.hy926 cell cultures with and without addition of TNF analyzed by qPCR and flow cytometry. Ea.hy926 cells were grown under 2D (*A *and *B*) and 3D (*C *and *D*) conditions for 18 h to form a monolayer or spheroids, and then TNF was added to a concentration of 25 ng/mL. After 5 h, a portion of the cultures was used for the generation of mRNA, cDNA synthesis, and qPCR (*A *and *C*). Parallel cultures were incubated for 36 h and analyzed by flow cytometry after staining with antibodies to VEGFR-2 and ICAM-1 (*B *and *D*). The statistically significant difference (> 0.05) is indicated by bars. The data are presented as a relative gene expression (RGE) (*A *and *C*). RGE was calculated by the formula RGE = 2–ddCt [40], where 2D cultures with TNF were compared to control without TNF, and 3D cultures with TNF were compared to control without TNF (*A*). Similarly, 3D was compared to 2D and without TNF (*C*). Cytometry data are presented as a ratio of MFI in a TNF-activated culture to that in the control without TNF (*B*) or MFI in a 3D culture to that in a 2D culture (*D*)

Early events in Ea.hy926 cultures after TNF activation were analyzed based on the expression of the* ICAM-1*, *VEGFR-2*, *GM-CSF*, and *IL-6 *genes evaluated by qPCR. The qPCR data are normalized to the expression of actin mRNA and presented as a relative gene expression (RGE) that is calculated by the formula RGE = 2^–ddCt^ [40]. This method assesses the change in the number of gene copies in TNF-activated 2D and 3D cultures compared to that in the control (*Fig. 5A*). It is also possible to compare gene expression under 3D and 2D conditions in the presence and absence of TNF (*Fig. 5C*). *Figure 5* compares the expression of VEGFR-2 and ICAM-1 in Ea.hy926 cell cultures without stimulation and after stimulation with TNF for 5 h, assessed by qPCR (*Fig. 5A, C*) and flow cytometry (*Fig. 5 B, D*). TNF significantly increased the synthesis of ICAM-1 mRNA both in 2D and 3D cultures (*Fig. 5A*), which correlated with the flow cytometry data (*Fig. 5B*). The effect of TNF was lower in 3D cultures. According to the qPCR data, expression of VEGFR-2 increased slightly, but reliably (*Fig. 5C*); in this case, the protein level evaluated by flow cytometry did not change. The difference in the data may be associated with non-optimal qPCR conditions (different length of the primers, *Table 1*). In any case, the effect of TNF on the expression of the ICAM-1 gene was significantly greater than on VEGFR-2.

Expression of the *GM-CSF *and *IL-6 *genes was analyzed in a similar manner. RNA was isolated 5 h after the addition of TNF. Parallel cultures were used to analyze the synthesis of proteins; the supernatant was harvested 30 h after activation by TNF.

**Fig. 6:**
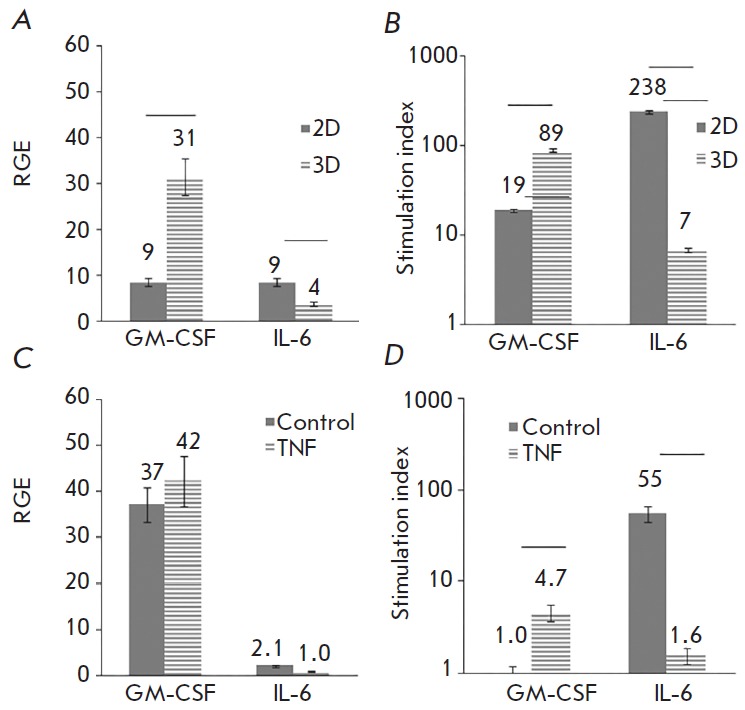
Production of GM-CSF and IL-6 in Ea.hy926 cell cultures with and without the addition of TNF analyzed by qPCR (*A *and *C*) and flow cytometry (*B *and *D*). Culture conditions and data processing were identical to those in* Figure 5*

*Figure 6* shows the level of spontaneous and TNF-induced synthesis of mRNA and the production of GMCSF and IL-6 proteins. Under the influence of TNF, both the synthesis of mRNA and the production of both proteins were enhanced. Stimulation of GM-CSF was more pronounced in 3D cultures, whereas stimulation of IL-6 was more effective in 2D cultures (*Fig. 6A, B*). Comparison of the efficiency of mRNA and protein synthesis in 2D and 3D cultures did not reveal differences in the level of gene expression (*Fig. 6C*). Spontaneous production of GM-CSF was identical under 2D and 3D conditions, whereas IL-6 production in 3D cultures was significantly higher. Upon stimulation with TNF, the differences shrank and higher production of both GM-CSF and IL-6 was observed in 3D cultures (*Fig. 6D*).

## CONCLUSION

For the first time, we have demonstrated that Ea.hy926 endothelial cells can be cultured on an anti-adhesive substrate under static conditions. In spontaneous Ea.hy926 cultures under 3D conditions, the production of both pro-inflammatory and anti-inflammatory factors is increased compared to that under 2D conditions, which enables a more detailed analysis when testing new therapeutic agents. TNF activation similarly affects Ea.hy926 cells cultured under 2D or 3D conditions, except for a 4- to 5-fold increase in the production of GM-CSF and IL-10 in 3D cultures. The most typical markers of Ea.hy926 cells are the adhesion molecule ICAM-1 and soluble factors IL-6, IL-8, TGF-β1, and IL-10. 3D cultures are easy to manipulate; they can be transferred onto new plates, e.g., 96-well plates, which enables one to study a panel of drugs in different dilutions. Confocal microscopy analysis does not require growing cells on glass slides. All these facts make the 3D culture of Ea.hy926 cells convenient for the screening of new anti-inflammatory and angiostatic drugs.

## Abbreviations

2D: two-dimensional conditions
3D: three-dimensional conditions
qPCR: quantitative polymerase chain reaction
ICAM-1: intercellular adhesion molecule 1
IFN: interferon
IL: interleukin
TNF: tumor necrosis factor
VCAM-1: vascular cell adhesion molecule 1
VEGF A: vascular endothelial growth factor A
VEGFR-2: vascular endothelial growth factor receptor 2
